# Effects of Nitric Oxide on Renal Proximal Tubular Na^+^ Transport

**DOI:** 10.1155/2017/6871081

**Published:** 2017-10-17

**Authors:** Nobuhiko Satoh, Motonobu Nakamura, Atsushi Suzuki, Hiroyuki Tsukada, Shoko Horita, Masashi Suzuki, Kyoji Moriya, George Seki

**Affiliations:** ^1^Department of Internal Medicine, The University of Tokyo Hospital, 7-3-1 Hongo, Bunkyo, Tokyo 113-8655, Japan; ^2^Department of Infection Control and Prevention, The University of Tokyo Hospital, 7-3-1 Hongo, Bunkyo, Tokyo 113-8655, Japan; ^3^Tokyo Gakugei University Health Service Center, 4-1-1 Nukuikita-machi, Koganei, Tokyo 184-8501, Japan; ^4^Yaizu City Hospital, Dobara 1000, Yaizu, Shizuoka 425-8505, Japan

## Abstract

Nitric oxide (NO) has a wide variety of physiological functions in the kidney. Besides the regulatory effects in intrarenal haemodynamics and glomerular microcirculation,* in vivo* studies reported the diuretic and natriuretic effects of NO. However, opposite results showing the stimulatory effect of NO on Na^+^ reabsorption in the proximal tubule led to an intense debate on its physiological roles. Animal studies have showed the biphasic effect of angiotensin II (Ang II) and the overall inhibitory effect of NO on the activity of proximal tubular Na^+^ transporters, the apical Na^+^/H^+^ exchanger isoform 3, basolateral Na^+^/K^+^ ATPase, and the Na^+^/HCO_3_^−^ cotransporter. However, whether these effects could be reproduced in humans remained unclear. Notably, our recent functional analysis of isolated proximal tubules demonstrated that Ang II dose-dependently stimulated human proximal tubular Na^+^ transport through the NO/guanosine 3′,5′-cyclic monophosphate (cGMP) pathway, confirming the human-specific regulation of proximal tubular transport via NO and Ang II. Of particular importance for this newly identified pathway is its possibility of being a human-specific therapeutic target for hypertension. In this review, we focus on NO-mediated regulation of proximal tubular Na^+^ transport, with emphasis on the interaction with individual Na^+^ transporters and the crosstalk with Ang II signalling.

## 1. Introduction

The proximal tubule is a key site for Na^+^ homeostasis via reabsorption of most of the water and solutes filtered by the glomerulus. Angiotensin II (Ang II), a powerful vasoconstrictor peptide, affects blood pressure via maintenance of Na^+^ homeostasis through its effects on the machinery for proximal tubular Na^+^ transport, including the apical Na^+^/H^+^ exchanger isoform 3 (NHE3), basolateral Na^+^/K^+^ ATPase (NKA), and the Na^+^/HCO_3_^−^ cotransporter (NBCe1) [1–5]. Interestingly, previous animal studies have confirmed that the effect of Ang II on proximal tubular transport is biphasic. Thus, low concentrations of Ang II increase transport activities, whereas high concentrations inhibit transport activities [6, 7]. However, whether the biphasic effect of Ang II could be reproduced in humans remained unclear.

Nitric oxide (NO) is a small gas molecule that diffuses freely through the plasma membranes of target cells and activates guanosine 3′,5′-cyclic monophosphate (cGMP) formation. Although NO was previously regarded as a toxic air pollutant, the discovery that it is identical to the previously recognised endothelium-derived relaxing factor [8, 9] has revealed its various physiological roles in the cardiovascular, neurologic, and immune systems [10–12]. Indeed, investigations have demonstrated the net natriuretic and diuretic effects and have indicated the inhibitory effect of NO on proximal tubular Na^+^ transport [13–15]. However, the reported effects were sometimes inconsistent or even controversial, partly because of the modification of NO effects by other regulatory and/or compensatory mechanisms occurring in a specific experimental condition. On the other hand, studies have shown that Ang II can mediate NO production via its specific cell-surface receptors (AT1 and AT2), suggesting a possible role of NO as a secondary messenger for Ang II-mediated regulation of proximal tubular transport [12, 16]. Therefore, the crosstalk between NO and Ang II signalling is an important factor to determine the effects of NO on proximal tubular transport. Here, we review NO-mediated regulation of proximal tubular Na^+^ transport, with emphasis on the interaction with individual Na^+^ transporters and the crosstalk with Ang II signalling.

## 2. Nitric Oxide Synthase (NOS): Isoforms and Distribution

NOS is a flavohemeprotein that catalyses the oxidation of l-arginine to l-citrulline with the production of NO, an important bioregulatory molecule in various physiological process such as neurotransmission, vasorelaxation, platelet aggregation, and immune responses [17–19]. The three known NOS isoforms are neuronal NOS (nNOS), endothelial NOS (eNOS), and inducible NOS (iNOS), and these show tissue-specific distribution and physiological functions [20–22].

nNOS is constitutively expressed throughout the central and peripheral nervous system, and it is associated with neuronal signalling. It produces low levels of NO, and its enzyme activity is strictly dependent on intracellular Ca^2+^ and calmodulin [23]. Similar to nNOS, eNOS generates relatively low levels of NO in the vascular endothelium and regulates vascular homeostasis in a Ca^2+^-dependent manner [24]. In contrast with these constitutive NOS enzymes, iNOS expression lacks cell specificity. Thus, it is not usually expressed but can be induced in almost all cell types on immune-related stimulation or gene transcription associated with bacterial lipopolysaccharides (LPSs), inflammatory cytokines, and other chemical mediators [25, 26]. Once induced, iNOS produces high levels of NO independently of the intracellular Ca^2+^ concentration [27], contributing to the pathophysiology of inflammatory disease and septic shock [25, 28].

## 3. Distribution of NOS in the Kidney

In the kidney, all three NOS isoforms are expressed at various locations along the nephron and are subjected to distinct control mechanisms [18, 29]. Immunostaining analysis has shown high expression of nNOS in the macula densa in all species, including humans [30, 31], suggesting an important role of nNOS in the juxtaglomerular apparatus and tubuloglomerular feedback (TGF) [32]. Further, the mRNA expression of nNOS has been reported in the thin limb of the loop of Henle and the medullary collecting duct [33]. eNOS is expressed widely in the epithelial cells of intrarenal vessels except in the venous system and regulates renal vascular tone [34]. In the tubular segments, high levels of eNOS mRNA have been reported in the proximal tubule, medullary thick ascending limb of the loop of Henle (mTAL), and collecting duct [24, 35].

iNOS is considered to have a wide distribution in the tubular epithelium. Generally, its expression is only induced after appropriate stimulation; however, in situ hybridisation studies have identified constitutive expression of iNOS mRNA in normal rat kidneys. iNOS mRNA has been detected in the S3 segment of the proximal tubule, mTAL, distal convoluted tubule, cortical collecting duct, and inner medullary collecting duct [36].

## 4. Physiological Functions of NO in Nephron Transport

It is well known that NO has a wide variety of physiological functions in the kidney based on the unique NOS population. For example, as a potent vasodilator, NO contributes to maintain a low vascular tone necessary for normal renal blood flow [37, 38]. On the other hand, NO released from the macula densa is involved in rennin secretion and TGF response via vasoconstriction of the afferent arterioles [39–41]. Besides the regulatory roles of NO in intrarenal haemodynamics and glomerular microcirculation,* in vivo* animal studies have shown the physiological importance of NO in renal Na^+^ transport [42, 43]. In these studies, infusion of NOS inhibitors into the kidney reduced water and Na^+^excretion, whereas the stimulation of NO production increased excretion. No significant change in the glomerular filtration rate (GFR) or renal blood flow (RBF) was observed with regard to the natriuretic and diuretic effects of NO, suggesting that NO could directly suppress nephron transport.

Considering the fact that NOS KO mice were significantly hypertensive and showed various cardiovascular abnormalities [44, 45], NO and NOS in nephron transport are possible key factors to elucidate the aetiology of Na^+^ retention and resultant hypertension. However, the functional significance of NO on individual nephron segments has recently been questioned [43, 45–47]. Despite the overall inhibitory effect of NO in nephron transport, several studies have demonstrated some controversial results for each nephron segment [43, 48–53]. Above all, the renal proximal tubule, which is the most important nephron portion for water and Na^+^ reabsorption, is intimately involved in NO-mediated renal Na^+^ transport regulation.

## 5. Effects of NO on Proximal Tubular Na^+^ Transport

**Figure 1:**
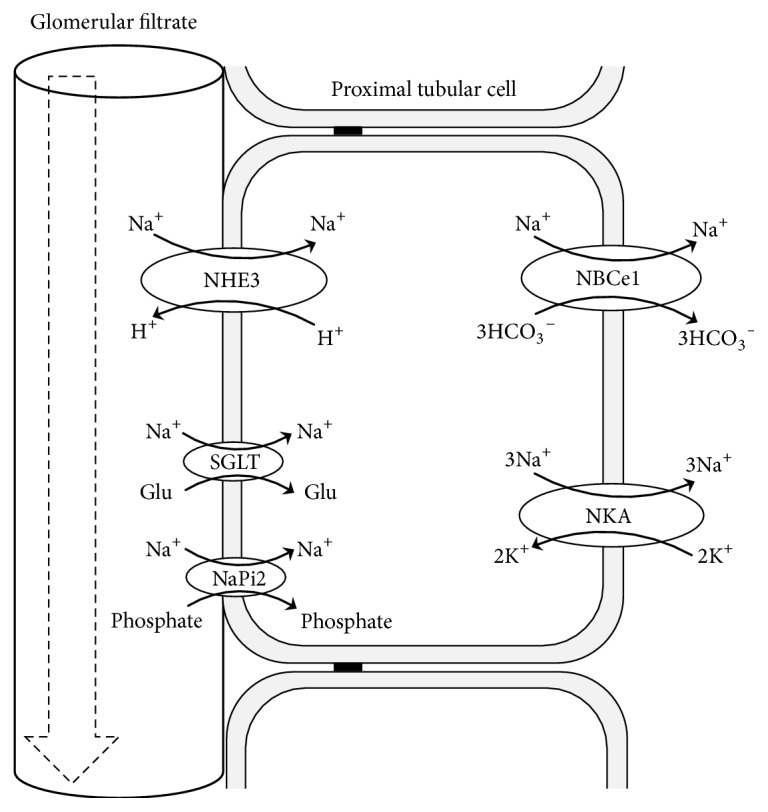
*Proximal tubular Na* ^+^ *reabsorption*. Active Na^+^ transport mediated by basolateral NKA provides the driving force for proximal tubular Na^+^ transport. Transporters in the apical membrane include NHE3, SGLT, and NaPi2. NHE3 is considered to be responsible for most of the Na^+^ reabsorption from the glomerular filtrate. NBCe1 at the basolateral membrane plays an important role not only in Na^+^ homeostasis but also in the systemic acid/base balance. NKA, Na^+^/K^+^ ATPase; NHE3, Na^+^/H^+^ exchanger isoform 3; NBCe1, Na^+^/HCO_3_^−^ cotransporter; Glu, glucose; SGLT, Na^+^/glucose cotransporter; NaPi2, Na^+^/phosphate cotransporter type 2.

Proximal tubules reabsorb approximately two-thirds of water and Na^+^ filtered in the glomeruli along with glucose, phosphate, amino acids and bicarbonates (HCO_3_^−^). Most of these solutes are carried via a series of Na^+^-coupled cotransporters or exchangers, which utilise the transmembrane Na^+^ gradient generated by NKA on the basolateral membrane [42]. Proximal tubule Na^+^ transport is thus primarily driven by NKA and predominantly accomplished by NHE3 on the apical membrane and NBCe1 on the basolateral membrane (Figure 1).

Although the effect of NO on proximal tubular Na^+^ transport is still controversial, a number of animal studies have reported its overall inhibitory effect. For example, in rat studies, the NO donor sodium nitroprusside (SNP) reduced proximal tubule fluid absorption (*Jv*), whereas the NO inhibitor NG-monomethyl-L-arginine (l-NAME) increased *Jv*, suggesting the inhibitory effect of NO on proximal tubular Na^+^ reabsorption [50, 54]. Further, using the microperfusion technique, Vallon et al. reported higher fluid and chloride reabsorption rates in the proximal tubules of nNOS KO mice than in the tubules of wild-type mice [51]. In contrast, Wang observed that both rats and mice treated with l-NAME presented significant diuresis and natriuresis due to decreased *Jv* and renal tubular absorption of HCO_3_^−^ (*J*_HCO3_), indicating the stimulatory effect of NO on proximal tubular transport [52, 53]. Moreover, they suggested that nNOS and iNOS could directly stimulate proximal tubular Na^+^ transport on the basis of lower *Jv* and *J*_HCO3_ in respective gene KO mice [55].

One possible reason for the discrepancy in the results on the effects of NO on proximal tubular transport is that the drug effects are modified by other regulatory and/or compensatory mechanisms occurring in a specific experimental condition. For example, effects of l-NAME on proximal tubular transport can be inhibited by denervation of the kidney, suggesting that the results reflect the differences in neural activity [54]. Additionally, the systemic administration of NOS inhibitors has been reported to increase renal perfusion pressure and induce flow-dependent changes in *Jv* and *J*_HCO3_ [56]. Furthermore, KO mice studies involve the influence of removal of the NOS gene from not only the kidney but also all from other tissues, which can modify a variety of regulatory mechanisms for proximal tubular transport, such as hormonal regulation, renal autoregulation, and neural regulation. Therefore, it is unclear whether the data obtained from these* in vivo *studies truly reflect the direct effect of NO on specific nephron segments. To delineate the influence of such interaction and regulatory mechanisms,* in vitro* microperfusion studies should be performed.

## 6. NO as a Potential Mediator of Ang II

Ang II, a powerful effector of the renin-angiotensin system, controls blood pressure partially by regulating water and Na^+^ homeostasis [57–59]. Its various effects are mediated by the specific cell-surface receptors AT1 and AT2. The AT1 receptor is ubiquitously expressed and mediates most of the physiological functions of Ang II, including vasoconstriction, stimulation of aldosterone release and sympathetic nerve activity, promotion of cell growth, and inflammation, whereas the AT2 receptor functionally antagonises the effects of the AT1 receptor [60, 61].

Studies have provided evidence of functional interaction between Ang II and NO in the vascular system, where Ang II regulates the NO/cGMP signalling pathway both by the AT1 and by AT2 receptors. Ang II, via the AT2 receptor, stimulates bradykinin-dependent NO production [62]. In addition, via AT1 receptors, it also triggers the Ca^2+^/calmodulin-dependent eNOS activation in bovine endothelial cells [63]. Then Ang II-induced NO exerts a vasodilating effect through the NO/cGMP signalling pathway to protect against Ang II vasoconstriction. Conversely, long-term infusion of Ang II is known to decrease NO bioavailability via the AT1 receptor by increasing NAD(P)H oxidase-mediated vascular superoxide production [64]. Further, the superoxide also reduces the activity of soluble guanylyl cyclase, inhibiting the NO/cGMP signalling pathway. Such functional interactions between Ang II and NO are regulated precisely at multiple levels, and the imbalance between Ang II and NO is considered to be the aetiology of many cardiovascular diseases including hypertension, atherosclerosis, and congestive heart failure.

In the proximal tubule of mice, rats, and rabbits, Ang II regulates Na^+^ transport in a biphasic manner such that low concentrations (picomolar to nanomolar) stimulate and high concentrations (nanomolar to micromolar) inhibit reabsorption [3, 6, 7, 65]. Although the responsible receptors had been debated [4, 6, 66, 67], studies using isolated proximal tubules from KO mice have demonstrated that the biphasic effect of Ang II on Na^+^ transport is predominantly mediated via the AT_1A_ receptor, an isoform of the AT1 receptor [3, 65]. With regard to the stimulatory effect of Ang II, the involvement of the activation of protein kinase C (PKC) and/or the decrease in the intracellular cAMP concentration, which can activate the extracellular signal-regulated kinase (ERK) pathway, has been suggested [4, 68, 69]. On the other hand, it has been reported that activation of NO and its downstream effector cGMP by Ang II mediates the inhibitory effect by acting on the machinery for proximal tubular Na^+^ transport, NKA, NHE3, and NBCe1 [5, 70–72].

### 6.1. NKA

NKA is an active transporter on the basolateral membrane, which pumps Na^+^ from the cytoplasm to the interstitium against its concentration gradient, supplying the driving force for proximal tubular Na^+^ reabsorption. The impact of the NO/cGMP pathway on NKA activity has been observed in several cell lines. In cultured mouse proximal tubular cells (MCT cells), Guzman et al. found that endogenous NO/iNOS stimulation by LPS and interferon-gamma decreased the catalytic activity of ouabain-sensitive ATPase, consistent with the inhibitory effect of NO on NKA [73]. Further, in opossum kidney cells, NKA activity was inhibited by not only NO but also by a cGMP analogue, suggesting the possible presence of the NO/cGMP pathway in the regulation of proximal tubular NKA [74, 75]. In accordance with the data from cell experiments, Zhang et al. proposed the presence of crosstalk between the NO/cGMP pathway and Ang II signalling in rat proximal tubules [76]. Ang II stimulates both NKA activity and NO synthesis via the AT1 receptor at a low concentration, and as the peritubular concentration of Ang II rises, NO/cGMP signalling inhibits NKA activity, which is partially attributed to the biphasic regulation of Ang II for proximal tubular Na^+^ transport. In contrast, NO was shown to have no impact on NKA activity in LLC-PK1 cells (another cell line derived from pig kidney) [74, 77]. Taken together, these data suggest a definite inhibitory effect of NO/NOS on proximal tubular NKA; however, future studies are required to investigate the cell-specific heterogeneity in the regulation of NKA activity via the NO/cGMP signalling pathway.

### 6.2. NHE3

NHE3, the epithelial isoform of the Na^+^/H^+^ exchanger, is abundantly expressed in the apical membrane of renal proximal tubules and is responsible for most of the Na^+^/H^+^ change activity in the region [78–80]. In support of* in vivo* data that demonstrated NO-induced natriuresis, NO has been shown to inhibit NHE3 activity in the proximal tubule [81–83]. Roczniak et al. first investigated the effect of NO on rabbit proximal tubules by using the NO donor sodium nitroprusside (SNP) and concluded that NO inhibits Na^+^/H^+^ exchange activity via cGMP elevation [83]. In addition, it has been reported that chronic blockade of NO synthesis by l-NAME results in increased expression of NHE3, suggesting the regulatory role of endogenous NO in the expression of Na^+^ transporters in the kidneys [84].

The biphasic effect of Ang II via the AT1 receptor on proximal tubular NHE3 activity has been observed in several animal studies [6, 85, 86]. It has been suggested that while stimulation of NHE3 at a low Ang II concentration is complicated in the PKC, PKA, PI3 kinase, or phospholipase C (PLC)-calmodulin pathway [1, 87–89], the inhibitory effect of a high Ang II concentration is mediated by the activation of the cGMP/cGMP-dependent protein kinase (PKG) pathway [1].

### 6.3. NBCe1

NBCe1 is predominantly expressed in the basolateral membrane of renal proximal tubules, and it mediates most of the Na^+^-coupled HCO_3_^−^ cotransport, playing a pivotal role in Na^+^ homeostasis and systemic acid/base balance [90, 91]. Similar to NKA and NHE3, microperfusion studies demonstrated that the biphasic effect of Ang II on NBCe1 is observed in mouse and rat proximal tubules [3, 69, 92]. Further, our recent study revealed that the NO/cGMP/cGMP-dependent kinase II (cGKII) pathway mediates the inhibitory effect of Ang II on mouse proximal tubular NBCe1. Thus, the inhibitory effect of Ang II was lost in proximal tubules of cGKII-KO mice, and neither a SNP nor a cGMP analogue phosphorylated ERK. The overall data on NKA, NHE3, and NBCe1 obtained from animal studies support the inhibitory effect of NO on proximal tubular Na^+^ transport.

## 7. Human-Specific Effects of NO and Ang II on Proximal Tubular Na^+^ Transport

The effects of NO and Ang II on human proximal tubular transport have been widely discussed. Previous studies in humans demonstrated that systemic administration of NOS inhibitors decreased the fractional excretion of Na^+^ (FE_Na_) and lithium (FE_Li_), which was consistent with the inhibitory effect of NO on proximal tubular Na^+^ reabsorption [13–15]. However, it has been reported that the NO derivative failed to induce natriuresis [93] and was associated with Na^+^ retention and plasma volume expansion in humans [94], suggesting the presence of human-specific NO effects. Moreover, Rosenbaek et al. recently confirmed that sodium nitrate also failed to induce natriuresis in humans [95].

**Figure 2:**
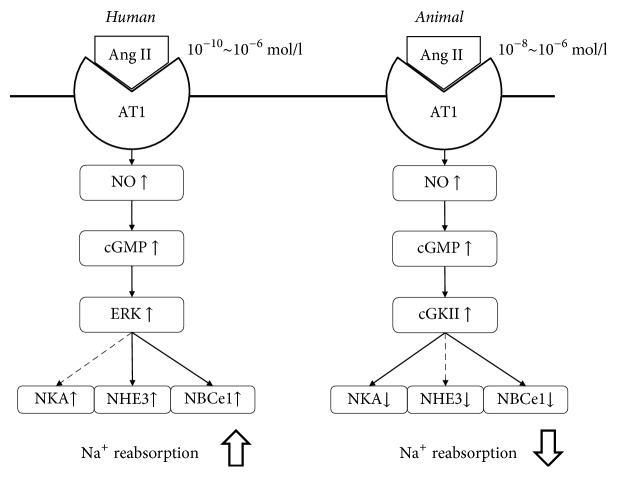
*Species difference in Ang II-mediated regulation of proximal tubular Na* ^+^ *transport*. In humans, Ang II dose-dependently stimulates the NO/cGMP pathway via the AT1 receptor over a wide range of concentrations (from low to high). The signalling cascade phosphorylates ERK, resulting in the stimulation of proximal tubular Na^+^ reabsorption, although the interplay between Ang II and NO signalling pathway on the NKA activity is still unconfirmed. In animal proximal tubules (mice, rats, and rabbits), a high concentration of Ang II also stimulates the NO/cGMP pathway, but the subsequent signalling cascade activates cGKII to exert inhibitory effects on Na^+^ reabsorption. As for NHE3, the inhibitory effect of Ang II is involved in the cGMP-dependent protein kinase, which is independent of the NO/cGMP pathway. Ang II, angiotensin II; AT1, angiotensin II receptor type 1; NO, nitric oxide; cGMP, guanosine 3′,5′-cyclic monophosphate; ERK, extracellular signal-regulated kinase; cGKII, cGMP-dependent protein kinase type II; NKA, Na^+^/K^+^ ATPase; NHE3, Na^+^/H^+^ exchanger isoform 3; NBCe1, Na^+^/HCO_3_^−^ cotransporter.

Interestingly, our functional analysis of isolated proximal tubules obtained from nephrectomy surgery demonstrated that Ang II dose-dependently stimulated human renal proximal tubular transport via ERK phosphorylation through the NO/cGMP pathway [92]. Thus, Ang II in human proximal tubules stimulates basolateral NBCe1 activity in a monophasic manner, unlike in animal proximal tubules. Furthermore, in luminal perfused human proximal tubules, we found that Ang II dose-dependently stimulated apical NHE3 activity and *J*_HCO3_. The exact reasons for such difference among species in the regulation of proximal tubular Na^+^ transport associated with the NO/cGMP pathway (Figure 2) are still unknown, but a high peritubular Ang II concentration [96], via its monophasic effect, would be more strongly associated with human hypertension [97].

## 8. Conclusion

NO plays a fundamental role in the regulation of proximal tubular Na^+^ transport. Although the direct effect of NO on proximal tubular transport has been intensely debated, the crosstalk with Ang II signalling and the interaction with Na^+^ transporters are possible key factors in solving this issue. The species difference in NO-mediated Ang II function is noteworthy. Our recent study identified human-specific monophasic regulation of proximal tubular transport through crosstalk between NO and Ang II signalling. Although its physiological significance is still unknown, the newly identified pathway that mediates the dose-dependent stimulatory effect of Ang II is expected to be a human-specific therapeutic target of hypertension. Future studies are necessary to clarify whether such a phenomenon is reproduced in other nephron segments, which has not yet been fully investigated.
